# Slum Health: From Understanding to Action

**DOI:** 10.1371/journal.pmed.0040295

**Published:** 2007-10-23

**Authors:** Alon Unger, Lee W Riley

## Abstract

The defining physical and legal characteristics of slums profoundly affect the health of these communities and may also serve as potential targets for immediate intervention.

*“Slums are a manifestation of the two main challenges facing human settlements development at the beginning of the new millennium: rapid urbanization and the urbanization of poverty.”*

Anna Kajumulo Tibaijuka, Executive Director, United Nations Human Settlements Programme [1]

*“Jo lives—that is to say, Jo has not yet died—in a ruinous place, known to the like of him by the name of Tom-all-Alone. It is a black, dilapidated street, avoided by all decent people…Now, these tumbling tenements contain, by night, a swarm of misery…As, on the ruined human wretch, vermin parasites appear, so, these ruined shelters have bred a crowd of foul existence that crawls in and out of gaps in walls and boards; and coils itself to sleep, in maggot numbers, where the rain drips in; and comes and goes, fetching and carrying fever…”*

Charles Dickens, *Bleak House* [2]

This year, 2007, marks the first time in human history that the majority of the world's population will live in cities [3]. The United Nations (UN) projects the world's urban population to grow by 2 billion before 2030. More than 90% of this growth will take place in the least developed countries [4], and will be concentrated in the bleakest parts of the city—human settlements known as slums. Already nearly a third (32%) of the world's population and more than three-fourths (78%) of the least developed countries' urban population live in slums [1]. Today's slums are unprecedented in their sheer magnitude, their rapidity of growth, and their worldwide distribution [1,5]. They represent a fundamental transformation of the physical and social environment of urban life and human health.

Like Dickens' Tom-All-Alone, slums are synonymous with squalid living conditions. A visit to the *favelas* of Rio de Janeiro, the shantytowns of Nairobi, or the *jhopadpatti* of Mumbai shows that a slum, by any name, is an unhealthy place to live (Table 1, Figures 1 and 2; for a virtual tour of one such slum, see: http://www.amref.org/index.asp?PageID=432). Many health outcomes are worse in slums than in neighboring urban areas or even rural areas [6–8]. Moreover, the formal health sector encounters slum residents only when they develop late-stage complications of preventable chronic diseases, as we have described elsewhere [9]. This takes a costly toll on these neglected communities and already limited health care resources. In this essay, we show that the defining physical and legal characteristics of slums profoundly affect the health of these communities and may also serve as potential targets for immediate intervention.

**Table 1:**
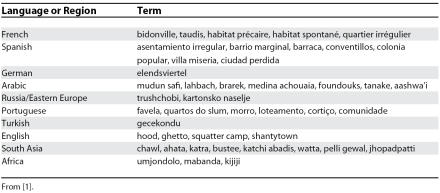
Slums Go By Many Names

**Figure 1:**
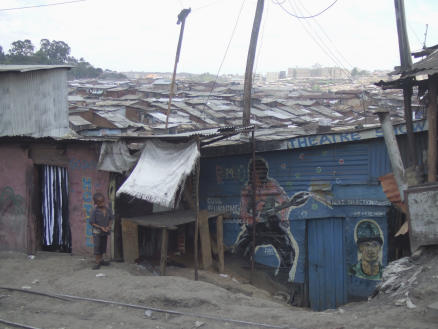
Kibera Shantytown in Nairobi, Kenya Home to nearly 1 million residents on the outskirts of Nairobi, Kibera is the world's second largest slum. Photo by Alon Unger. For a virtual tour, see: http://www.amref.org/index.asp?PageID=432

**Figure 2:**
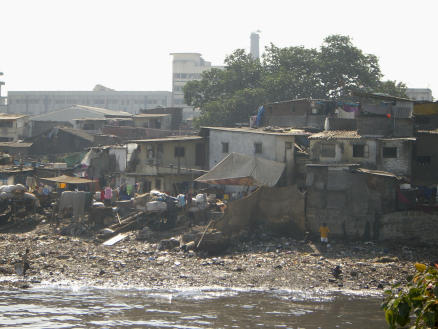
A *Jhopadpatti* in Mumbai, India There are 6.7 million slum dwellers among Mumbai's 12 million residents. In the Ganesh Murthy Nagar slum in the Colaba district, one resident noted, “We had one small, smelly toilet for a population of 10 000. Women suffered the most because they had to relieve themselves in the open, and could do so only in the early mornings or after dark” [43]. There are regular outbreaks of diarrheal diseases, leptospirosis, malaria, and dengue in Mumbai's slums [4]. Photo by Lee Riley.

## Defining Slums and the Challenge of Slum Health

In 2002, the UN operationally defined slums as those communities characterized by: insecure residential status, poor structural quality of housing, overcrowding, and inadequate access to safe water, sanitation, and other infrastructure [10] (Table 2). The 2003 UN report, *The Challenge of Slums,* is the most comprehensive account of the demographic and socioeconomic indicators of slums worldwide [1]. It details not only the high concentration of poverty and substandard living conditions in slums, but also the insecurity of tenure and marginalization from the formal sector, including basic health services.

**Table 2:**
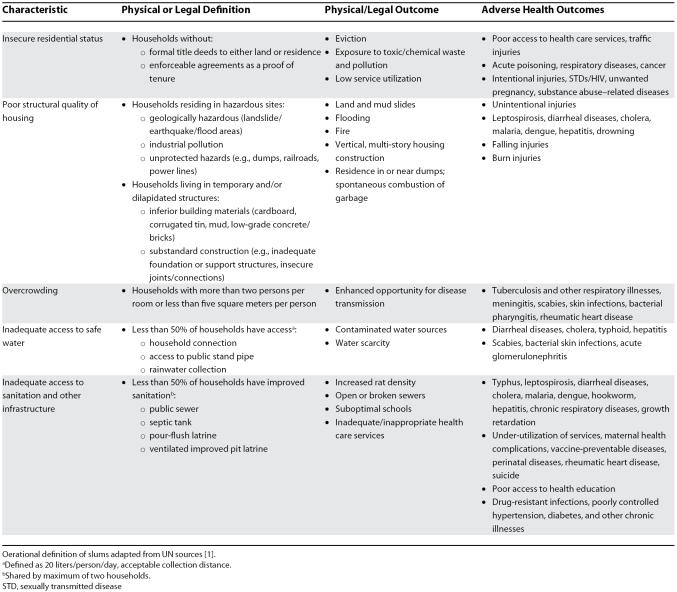
UN Operational Definition of Slums and the Physical, Legal, and Adverse Health Outcomes

## Conditions of Slum Life and Health—Using the UN Operational Definition

Slums are areas of “concentrated disadvantage” [11]. The physical and legal characteristics enumerated by the UN are intimately related with population composition and dynamics, social environment, poverty, and marginalization. Health comparisons of rural versus urban areas, or emphasis on the urban health “penalty” or “advantage,” do not highlight the specific health determinants of slums [12]. Here, we use the UN operational criteria to show that the conditions of slum life have a direct impact on the health and well-being of these communities (Table 2).

### Insecure residential status

The lack of secure land or housing tenure forces residents to occupy unused or undesirable land. For example, between 1991 and 1997, 1.5 million and 1 million people were evicted from central areas of Shanghai and Beijing, respectively [13]. Such dislocations out of the city center force large numbers to commute longer distance to their original place of work, where they brave the road, by foot or in overcrowded and dangerous vehicles, putting themselves at risk for road-traffic injuries [14]. Slum locations may be unused or undesirable because of their hazardous geography, such as landslide- or flood-prone areas, or unsafe or polluted environments. Moreover, their residential status limits their ability to fight for the right to a safe environment. In 1984, the accidental release of methyl isocyanate from a pesticide factory in Bhopal, India killed more than 20,000 slum residents; the factory was built after the settlement had already been in existence [15]. Even in the United States, Hurricane Katrina unmasked the vulnerability of residents of poor neighborhoods in flood-prone areas and also the neglect of this population by political institutions [16,17].

### Poor structural quality of housing

Slum housing is densely packed and poorly built with substandard or even flammable materials. Houses built against hillsides are subject to landslides during heavy rain, and inferior building standards cause many thousands of deaths from earthquakes, especially where urbanization and poverty collide [18]. In Bam, Iran, poor structural quality of housing played a major factor in the earthquake-related deaths of 32,000 people in 2003. Days earlier, an earthquake of a similar magnitude killed only two people in California. The built environment is also directly related to accidental injuries, such as falls and burns [19]. Physical characteristics of slums not only magnify the consequences of natural or man-made disasters, but also hinder rescue efforts [20].

### Overcrowding

Slum dwellings have high occupancy rates in all-purpose rooms. Cooking, sleeping, and living with 13.4 people per 45 m^2^ room, as in the slums of Kolkata, India [21], places residents at risk of respiratory infections, meningitis, and asthma [22,23]. In Manila, the Philippines, children living in squatter settlements are nine times more likely than other children to have tuberculosis (TB) [24]. Epidemic-prone infections like pertussis cluster in areas of urban poverty [25], and overcrowding may even fuel potentially emerging epidemic diseases like SARS or influenza [26]. Crowding is also associated with rheumatic heart disease, a chronic and debilitating disease facilitated by increased transmission of group A Streptococcus pyogenes infections and lack of early treatment [27].

### Inadequate access to safe water

Poor water quality is a leading cause of morbidity and mortality worldwide and a defining danger of living in slums [28]. Many life-threatening infectious diseases are associated with contaminated water in slums, such as cholera and hepatitis (Table 2) [29]. Lack of access to water also restricts water intake, sources for infant formula or cooking, bathing and personal hygiene. Infrequent bathing is associated with scabies and bacterial skin infections, a subset of which (i.e., group A streptococcus) can lead to acute glomerulonephritis [30].

### Inadequate access to sanitation and other infrastructure

The lack of infrastructure affects all aspects of life, including waste collection and sewers, public transportation, policing, education, and electricity supply. Five million slum residents live without toilets in Mumbai; if each person defecates half a kilogram per day, 2.5 million kilograms of human waste contaminate their environment each day [31]. While diseases like leptospirosis in developed countries generally occur as a result of recreational water sports [32], 95% of severe manifestations of this disease in Salvador, Brazil occur in slum residents living in areas with close proximity to open sewers and high rat density [33]. Slums are also excluded from the benefits of formal policing, and young men in the *favelas* of Brazil are up to five times more likely to die from homicide than their urban counterparts [34]. Violence associated with drug traffic between gangs or with the police create unsafe conditions for all residents and pose a major barrier to provision of public health interventions [35]. Violence towards women is also associated with the absence of basic services like street lighting [36].

Available health services are often comprised of an inconsistent patchwork of public, private, and charity-based providers. Inadequate or inappropriate care at these places permits the progression of preventable diseases, such as hypertension and diabetes, and increases the risk of drug-resistant infections, such as multidrug-resistant TB [37]. Vaccination coverage in slums is markedly lower than in other urban areas due to inadequate infrastructure and a lack of community awareness and mobilization [38]. Appropriate interventions and treatments are only effective once provided in the context of accessible and utilized health care services.

## Meeting the Challenge of the Slums

The determinants of slum health are too complex to be defined by any single parameter. Yet, they arise from a common physical and legal pedigree that concentrates the ill effects of poverty, unhealthy environments, and marginalization from the formal sector. The promotion of urban health in the 21st century must take neighborhood-centered as well as person-centered approaches. We recognize that broad economic, social, and political forces play an important role in the creation and growth of slums, and addressing these forces will take time. However, we represent clinicians and public health specialists, and therefore, our approach focuses on immediate solutions that can dramatically improve health and health disparities (Box 1).

**Box 1. Meeting the Challenge of Slums**

**Gather data on slum disease burden and intra-urban health disparities**

- Establish routine disease surveillance within slum communities or at local clinics
- Ensure a safe environment for disease surveillance and reporting of illegal or informal residential status, without fear of reprisal
- Organize community planning groups for health care prioritization made up of community leaders and representatives of local health professionals, high-risk groups, community-based organizations, and nongovernmental organizations
- Develop new analytical framework for understanding health outcomes in slums, including new metrics for disease burden estimates based on slum-specific social and physical parameters

**Identify and target relevant and modifiable conditions of slums life**

- Focus on slum-specific health care needs, which may be very different than those in neighboring urban areas
- Target immediately modifiable health risks, such as diverting sewage or run-off, waste disposal, installing public lighting, providing soap and hygiene education, and improving traffic safety
- Involve auxiliary health care providers, such as private pharmacies and traditional healers

**Take action**

- Use existing structures and social capital in slums, such as community groups or religious institutions, and involve residents in design and provision of services
- Engage in multisectoral interventions, including professionals from urban planning, public works, engineering, and health sectors
- Advocate for patients through public advocacy, political institutions, and reporting study results in internationally distributed professional journals

### Gather data on slum disease burden and intra-urban health disparities

Accurate health statistics in slums are difficult to obtain, and health statistics rarely report intra-urban differences. The inability to collect or analyze detailed urban health data masks gross health disparities within cities. Currently, most slum disease burden and mortality data are based on clinic, hospital, or national mortality registry data. This grossly underestimates the underlying medical conditions, such as hypertension, that give rise to the complications observed by the formal health sector, such as stroke [9]. The absence of detailed or accurate data limits the ability of officials to detect health threats or appropriately allocate resources. Prompt identification of local health concerns is the first step in any intervention.

There is a pressing need for a new analytic framework to understand health in slums. Standard health metrics, such as disability-adjusted life years (DALYs) lost, do not account for the context in which diseases occur. The DALY for TB in a 25-year-old man in New York City is different than that in a 25-year-old man in the Dharavi slum of Mumbai. Accepted indicators of socioeconomic status (e.g., income, education, occupation) are inadequate in areas of generalized poverty and informal residence or work [39]. The social gradient within slums may be better indicated, for instance, by the difference in building materials (i.e., mud versus brick) or number of meals per day. Slums are complex, and our efforts must match this complexity. Then we can better focus scarce resources on the most modifiable and relevant factors for improving health. This will save time, money, and lives.

### Targeting relevant and modifiable conditions of slum life

Slum-specific information may reveal that health priorities in slums should be very different than national or even local urban ones. Improving health status may require the simple acts of closing open sewers to limit diarrheal disease, lighting footpaths to deter violence, constructing barriers to prevent falling injuries, or diverting run-off or reinforcing dams to lessen loss of property and life from heavy rains. Some priorities, like addressing high rates of HIV in slums, may be the same as for other communities; however, the solutions must be slum-specific [40]. These efforts can be made without awaiting poverty alleviation. Intervention trials in poor urban/slum areas have already demonstrated that hand-washing with soap can reduce the risk of diarrheal diseases by up to 47% and could save a million lives [41]. Compelling data like these can be used to advocate for improved sanitation infrastructure and basic hygiene supplies.

### From understanding to action

Health officials, doctors, and public health specialists can take advantage of existing structures and social capital in slums. Dense populations may not only pose a risk, but also an opportunity—to efficiently reach a large, vulnerable proportion of the population. We can minimize the lack of access to health services through innovative programs, such as school-based vaccination or outreach via churches, temples, and mosques. This means finding people where they congregate, be it bars in Venezuela or dance halls in Kenya. In particular, private pharmacies are central to health care in slums [42] and can play an important role in monitoring chronic diseases (e.g., hypertension and diabetes) and delivering health education in slums.

It is more important than ever to enlist residents of slums as partners. In Mumbai, residents are instrumental in managing community toilets in a nationwide project supported by the World Bank [43]. In Rio de Janeiro, community members educate their neighbors about HIV infection and hand out condoms in markets [35]. Involving residents is also an important step in redressing the social exclusion, inequity, and disempowerment that characterize their situation.

Interventions to improve the health of slum dwellers are not cutting-edge science. Effective interventions involve not only treating disease but also addressing the underlying social and living conditions of slums. Many solutions will require significant multisectoral effort and resource mobilization, which may be beyond our traditional role as health professionals. This will require us to be students of problems, not disciplines, and to work closely with urban planners, engineers, and politicians to make the necessary changes. Health professionals can also make important contributions as civic leaders when they organize neighborhood associations and resident advocacy groups, or act themselves to represent the billion unheard voices of slum dwellers.

## Conclusion

We describe the challenges to good health and health care presented by the physical and legal characteristics of slums. It will take time to address underlying political, economic, and social forces that create and perpetuate slums. Yet many interventions and services can be implemented immediately with life-saving effect. Our approach to slum health is neither comprehensive nor exclusive. It is our simple goal to direct the attention and efforts of health professionals to the characteristics of slum life that profoundly affect health. Together we can move from understanding to action, and improve the lives of people who live in slums right now.

## Abbreviations

DALY: disability-adjusted life year
TB: tuberculosis
UN: United Nations

## References

[pmed-0040295-b001] UN-HABITAT (2003). The challenge of the slums: Global report on human settlements.

[pmed-0040295-b002] Dickens C (1853). Bleak House.

[pmed-0040295-b003] United Nations Population Division (2003). World urbanization prospects: The 2003 revision. http://www.un.org/esa/population/publications/wup2003/WUP2003Report.pdf.

[pmed-0040295-b004] United Nations Population Division (2002). World urbanization prospects: The 2001 revision. http://www.un.org/esa/population/publications/wup2001/WUP2001report.htm.

[pmed-0040295-b005] Davis M (2006). Planet of slums.

[pmed-0040295-b006] Szwarcwald CL, Andrade CL, Bastos FI (2002). Income inequality, residential poverty clustering and infant mortality: A study in Rio de Janeiro, Brazil. Soc Sci Med.

[pmed-0040295-b007] Sclar ED, Garau P, Carolini G (2005). The 21st century health challenge of slums and cities. Lancet.

[pmed-0040295-b008] Fotso JC (2006). Child health inequities in developing countries: Differences across urban and rural areas. Int J Equity Health.

[pmed-0040295-b009] Riley LW, Ko AI, Unger A, Reis MG (2007). Slum health: Diseases of neglected populations. BMC Int Health Hum Rights.

[pmed-0040295-b010] UN-HABITAT (2002). Defining slums: Towards an operational definition for measuring slums.

[pmed-0040295-b011] Vlahov D, Freudenberg N, Proietti F, Ompad D, Quinn A (2007). Urban as a determinant of health. J Urban Health.

[pmed-0040295-b012] Freudenberg N, Galea S, Vlahov D (2005). Beyond urban penalty and urban sprawl: Back to living conditions as the focus of urban health. J Community Health.

[pmed-0040295-b013] Zhang Y, Fang K (2004). Is history repeating itself? From urban renewal in the United States to inner-city redevelopment in China. J Plann Educ Res.

[pmed-0040295-b014] Ameratunga S, Hijar M, Norton R (2006). Road-traffic injuries: Confronting disparities to address a global-health problem. Lancet.

[pmed-0040295-b015] Dhara VR, Dhara R (2002). The Union Carbide disaster in Bhopal: A review of health effects. Arch Environ Health.

[pmed-0040295-b016] Nates JL, Moyer VA (2005). Lessons from Hurricane Katrina, tsunamis, and other disasters. Lancet.

[pmed-0040295-b017] McLellan F (2005). Hurricane Katrina: “A speaking sight”, or, washday in Durant. Lancet.

[pmed-0040295-b018] Jackson J (2006). Fatal attraction: Living with earthquakes, the growth of villages into megacities, and earthquake vulnerability in the modern world. Philos Transact A Math Phys Eng Sci.

[pmed-0040295-b019] Bartlett SN (2002). The problem of children's injuries in low-income countries: A review. Health Policy Plan.

[pmed-0040295-b020] Sapir D, Lechat M (1986). Reducing the impact of natural disasters: Why aren't we better prepared?. Health Policy Plan.

[pmed-0040295-b021] Kundu N (2003). Urban slum reports: The case of Kolkata, India.

[pmed-0040295-b022] Sharma S, Sethi GR, Rohtagi A, Chaudhary A, Shankar R (1998). Indoor air quality and acute lower respiratory infection in Indian urban slums. Environ Health Perspect.

[pmed-0040295-b023] Benicio MH, Ferreira MU, Cardoso MR, Konno SC, Monteiro CA (2004). Wheezing conditions in early childhood: Prevalence and risk factors in the city of Sao Paulo, Brazil. Bull World Health Organ.

[pmed-0040295-b024] Fry S, Cousins B, Olivola K (2002). Health of children living in urban slums in Asia and the near east: Review of existing literature and data. Environmental Health Project, U.S. Agency for International Development. http://www.ehproject.org/PDF/Activity_Reports/AR109ANEUrbHlthweb.pdf.

[pmed-0040295-b025] Siegel C, Davidson A, Kafadar K, Norris JM, Todd J (1997). Geographic analysis of pertussis infection in an urban area: A tool for health services planning. Am J Public Health.

[pmed-0040295-b026] Davis M (2005). The monster at our door: The global threat of avian flu.

[pmed-0040295-b027] World Health Organization (2004). Rheumatic fever and rheumatic heart disease. http://www.who.int/cardiovascular_diseases/resources/trs923/en/index.html.

[pmed-0040295-b028] World Health Organization (2003). Emerging issues in water and infectious disease. http://www.who.int/water_sanitation_health/emerging/emergingissues/en/.

[pmed-0040295-b029] UN-HABITAT (2003). Water and sanitation in the world's cities.

[pmed-0040295-b030] Heukelbach J, Wilcke T, Winter B, Feldmeier H (2005). Epidemiology and morbidity of scabies and pediculosis capitis in resource-poor communities in Brazil. Br J Dermatol.

[pmed-0040295-b031] Mehta S 2004 Maximum city: Bombay lost and found New York Alfred A Knopf.

[pmed-0040295-b032] Dziuban EJ, Liang JL, Craun GF, Hill V, Yu PA (2006). Surveillance for waterborne disease and outbreaks associated with recreational water—United States, 2003–2004. MMWR Surveill Summ.

[pmed-0040295-b033] Ko AI, Galvao Reis M, Ribeiro Dourado CM, Johnson WD, Riley LW (1999). Urban epidemic of severe leptospirosis in Brazil. Salvador Leptospirosis Study Group. Lancet.

[pmed-0040295-b034] National Academies Press (2003). Cities transformed: Demographic change and its implications for the developing world. http://www.nap.edu/catalog.php?record_id=10693.

[pmed-0040295-b035] Loewenberg S (2005). Tackling the causes of ill health in Rio's slums. Lancet.

[pmed-0040295-b036] Krishnakumar A (2003). A sanitation emergency. Focus. http://www.hinduonnet.com/fline/fl2024/stories/20031205002510100.htm.

[pmed-0040295-b037] Bates I, Fenton C, Gruber J, Lalloo D, Lara AM (2004). Vulnerability to malaria, tuberculosis, and HIV/AIDS infection and disease. Part II: Determinants operating at environmental and institutional level. Lancet Infect Dis.

[pmed-0040295-b038] Agarwal S, Bhanot A, Goindi G (2005). Understanding and addressing childhood immunization coverage in urban slums. Indian Pediatrics.

[pmed-0040295-b039] Ompad DC, Galea S, Caiaffa WT, Vlahov D (2007). Social determinants of the health of urban populations: Methodologic considerations. J Urban Health.

[pmed-0040295-b040] Amuyunzu M, Okeng'o L, Wagura A, Mwenzwa E (2007). Putting on a brave face: The experiences of women living with HIV and AIDS in informal settlements of Nairobi. AIDS Care.

[pmed-0040295-b041] Curtis V, Cairncross S (2003). Effect of washing hands with soap on diarrhoea risk in the community: A systematic review. Lancet Infectious Diseases.

[pmed-0040295-b042] Amuyunzu-Nyamongo M, Nyamongo IK (2006). Health-seeking behaviour of mothers of under-five-year-old children in the slum communities of Nairobi, Kenya. Anthropology & Medicine.

[pmed-0040295-b043] Chinai R (2002). Mumbai slum dwellers' sewage project goes nationwide. Bull World Health Organ.

